# A High Molar Extinction Coefficient Mono-Anthracenyl Bipyridyl Heteroleptic Ruthenium(II) Complex: Synthesis, Photophysical and Electrochemical Properties

**DOI:** 10.3390/molecules16064615

**Published:** 2011-06-03

**Authors:** Adewale O. Adeloye, Peter A. Ajibade

**Affiliations:** Department of Chemistry, Faculty of Science and Agriculture, University of Fort Hare, P.M.B. X1314, Alice 5700, South Africa; Email: pajibade@ufh.ac.za (P.A.A.)

**Keywords:** Ru(II) complex, bipyridine, extended π-bond conjugation, spectroscopy, molar extinction coefficient, molecular aggregation

## Abstract

In our quest to develop good materials as photosensitizers for photovoltaic dye-sensitized solar cells (DSSCs), *cis*-dithiocyanato-4-(2,3-dimethylacrylic acid)-2,2'-bipyridyl-4-(9-anthracenyl-(2,3-dimethylacrylic)-2,2'-bipyridyl ruthenium(II) complex, a high molar extinction coefficient charge transfer sensitizer, was designed, synthesized and characterized by spectroscopy and electrochemical techniques. Earlier studies on heteroleptic ruthenium(II) complex analogues containing functionalized oligo-anthracenyl phenanthroline ligands have been reported and documented. Based on a general linear correlation between increase in the length of π-conjugation bond and the molar extinction coefficients, herein, we report the photophysical and electrochemical properties of a Ru(II) bipyridyl complex analogue with a single functionalized anthracenyl unit. Interestingly, the complex shows better broad and intense metal-to ligand charge transfer (MLCT) band absorption with higher molar extinction coefficient (λ_max_ = 518 nm, ε = 44900 M^−1^cm^−1^), and appreciable photoluminescence spanning the visible region than those containing higher anthracenyl units. It was shown that molar absorption coefficient of the complexes may not be solely depended on the extended p-conjugation but are reduced by molecular aggregation in the molecules.

## 1. Introduction

The quest for new materials that efficiently harvest solar light continues to be an important goal. Recently, considerable efforts have been focused on new photosensitizers, including ruthenium complexes [1,2,3,4,5,6,7,8,9,10] and organic dyes [11,12,13,14], in dye-sensitized nanocrystalline solar cells (DSSCs) since *cis*-dithiocyanato bis(4,4'-dicarboxy-2,2'-bipyridine) ruthenium(II) [Ru(dcbpy)_2_(NCS)_2_] (referred as N3) anchored on porous nanocrystalline TiO_2_ electrode has exhibited 10% light-to-electric power conversion efficiency. The efficiency of sensitization is critically dependent on electron injection from a photoexcited state of the dye into the conduction band of the semiconductor.

It has been shown that one of the best way to enhance both the absorption coefficient and red-shift of the metal-to ligand charge transfer (MLCT) band in a ruthenium-based photosensitizer was to extend the π-conjugation length of the colorant’s ancillary [15] or anchoring [16] ligands. Other classes of ligands such as carboxylated terpyridine and phenanthroline showed enhanced UV-Vis absorption over a broad range due to their large conjugated backbone structure. These ligands can be utilized as efficient light harvesting sensitizers as well [17]. Various studies have also shown that it is not an impossible task to increase the excited state lifetime of Ru(II) polypyridyl complexes based on polypyridyl ligands. However, if all the requirements are to be fulfilled and the driving force for further reactions be high enough, a new approach that does not stabilize the ^3^MLCT state but rather destabilized the ^3^MC state has to be developed [18,19].

Existing design strategies for the preparation of these advanced multicomponent molecules follow two principal methods. One approach exploits the use of metallosynthons bearing reactive functions such as triflates, halides and carbonyls as the intermediates for producing the final species with the expected features. In another approach, the ligand is first constructed and subsequently coordinated to the appropriate metal. In both cases, the quest for new and alternative approaches for easy building and organizing various photoactive partners around photoactive metals is one of the main aims of this field [20]. Some authors reported the synthesis of heteroleptic ruthenium complexes by extending the conjugation length of the ancillary ligand [21,22]. Such heteroleptic ruthenium complexes have a strong MLCT band and dye solar cells devices based on them display very good photovoltaic performance. In spite of this, the main drawback of these sensitizers is the lack of absorption in the red region of the visible spectrum and also relatively low molar extinction coefficient [23]. Many researchers have tried to overcome these shortcomings without significant success [24,25,26]. The molecular engineering of ruthenium complexes for TiO_2_-based solar cells presents a challenging task as several stringent requirements have to be fulfilled by the sensitizer and these are very difficult to be met simultaneously, including absorption of all the visible light and function as an efficient charge transfer sensitizer [27]. Our strategy in turning the excited state properties of the Ru(II) complexes is based on the new design of bidentate polypyridyl ligands by extending the p-system of the ligands using both unsaturated alkylene and polyaromatic molecules of which the anchoring ligand group carry carboxylic acid functionality which is to serve as linkage to the semiconductor nanocrystalline titanium dioxide in an application such as the dye-sensitized solar cells.

The emphasis of the present work is the synthesis of a new ruthenium(II) bipyridyl complex incorporating a single anthracenyl unit as substituent on a ligand, thus showing the effect in the reduction of molecular aggregation as found in the previous reports for functionalized oligo-anthracenyl Ru(II) phenanthroline complexes [28,29]. Herein, we present the synthetic methodology, photophysical, spectroscopical characterization, and the electroredox properties of the complex.

## 2. Results and Discussion

### 2.1. Chemistry

**Scheme 1:**
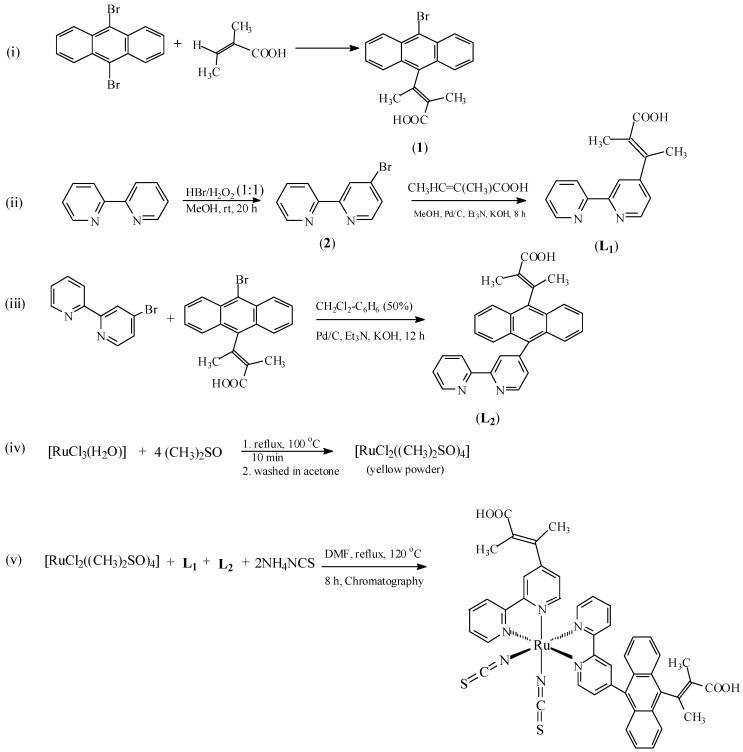
Synthetic pathways for Ligands **L_1_**, **L_2_** and [**RuL_1_L_2_(NCS)_2_**].

Scheme 1 (i-iii) show the stepwise synthetic pathways for **L_1_** and **L_2_**, and outline the chemistry of the present study. 9,10-Dibromoanthracene, 2,3-dimethylacrylic acid and 2,2'-bipyridine are the starting materials for **1** and **2**. 9-Bromo-10-(2,3-dimethylacrylic acid)-anthracene (**1**) was obtained when 9,10-dibromoanthracene and 2,3-dimethylacrylic acid were refluxed in a benzene/dichloromethane mixture under basic condition using triethylamine, potassium hydroxide and palladium carbide. Product **2** was obtained by the initial introduction of required bromine functions on bipyridine under anenvironmentally benign condition as reported by Vyas and co-workers [30]. **L_1_** and **L_2_** were synthesized following slight modifications of established procedures reported in the literature [29]. **L_1_** was obtained by one-step nucleophilic aromatic substitution reaction of the bromide group of product **2** with 2,3-dimethylacrylic acid. The synthesis of **L_2_**, essentially follows two major reaction steps: the initial synthesis of a 2,3-dimethylacrylic acid functionalized anthracenyl group followed by a palladium cross-catalyzed reaction of two halide compounds. This reaction was aided by the use of an equivalent volume ratio of dichloromethane-benzene as solvent to overcome the poor solubility of anthracenyl derivatives in common organic solvents such as methanol and chloroform. It was found that this reaction could be reversed by first reaction of the halogenated polypyridine with 9,10-dibromoanthracene and a subsequent dehydrohalogenation reaction with 2,3-dimethylacrylic acid. The synthesis of the metal precursor [RuCl_2_(dmso)_4_] [31], and the complex [RuL_1_L_2_(NCS)_2_] followed the general synthetic route shown in Scheme 1 (iv and v). Sequential substitution of the DMSO coordinating ligand from the metal precursor with **L_1_** and **L_2_** led to formation of intermediate complex [RuL_1_L_2_Cl_2_] which was not isolated. Final reaction was carried out with chloride exchange with thiocyanate group [32].

### 2.2. Infrared Spectra

**Figure 1:**
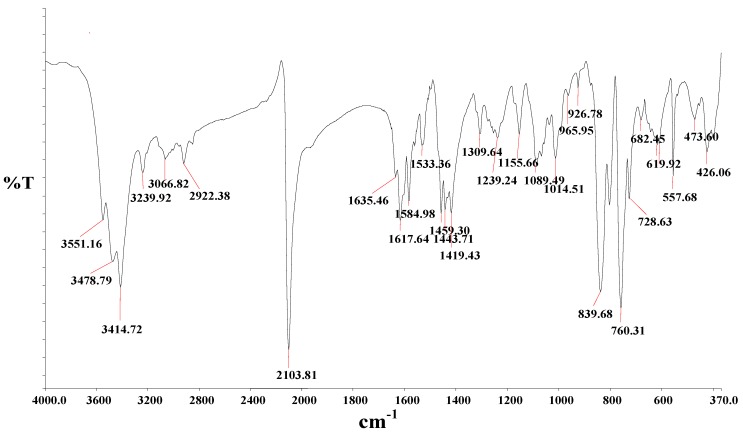
FT-IR spectrum of [**RuL_1_L_2_(NCS)_2_**] complex in KBr.

The FT-IR spectra of the starting materials, the ligands and the complex showed certain characteristic absorption bands that were compared and assigned on careful comparison. Due to structural similarities between the ligands, and the complex, a strong band in the region 3427–3054 cm^−1^ was found. This gave indication of the presence of an O-H group possibly from the carboxylic acid moiety. This band, however, shifted to higher frequency at 3551 cm^−1^ in the complex (Figure 1). The vibration frequency bands between 3478-3066 cm^−1^ may be due to presence of α,β-unsaturated carboxylic acid and/or aromatic C-H stretching characteristics of the molecules. The band at 2927 cm^−1^ shows the presence of C-H stretching of methyl groups. The complex shows a broad absorption frequency at 2103 cm^−1^ for stretch vibrational modes due to the N-coordinated υ(CN). This band is very close in intensity compared to the band at 839 cm^−1^, due to υ(CS). The bands at 1635 cm^−1^ and 1239 cm^−1^ were assigned to the υ(C=O) and υ(C–O) stretching of carboxylic acid groups, respectively.

The three bands at 1584, 1533 and 1443 cm^−1^ are due to ring stretching modes of the ligands. The band at 1309 cm^−1^ was assigned to the carboxylate symmetric υ(–COO–) of the carboxylic acid group. A comparison of the infrared spectra of **L_2_** and 9,10-dibromoanthracene showed that a strong vibrational band in the former was conspicuously absent in the latter, confirming the loss of C–Br bond and the formation of C–C bond linkages of the anthracenyl group. Furthermore, the C–C bond linkage between anthracene and bipyridine was affirmed by the absorption frequency at 805 cm^−1^. Peaks in the region 760 and 718 cm^−1^ demonstrate the existence of four adjacent hydrogen atoms common to **L_2_** and complex [RuL_1_L_2_(NCS)_2_]. All vibrational peaks in the region are found to be relatively weak and broad in the complex, which may be ascribed to the loss of crystallinity and the broad distribution of the anthracene chain length [33]. The weak absorption frequencies at 473 and 426 cm^−1^, respectively, show the coordination of nitrogen atoms of the ancillary ligands to ruthenium central metal atom [34].

### 2.3. ^1^H and ^13^C-NMR Spectroscopic Studies

The aromatic region of the ^1^H-NMR spectrum of **L_1_** gave six peaks at 8.66 (d, 1H), 8.41 (d, 1H), 7.86 (dd, 1H), 7.36 (1H), 1.73 (s, 3H), 1.66 (d, 3H) ppm. The ^1^H peaks are very similar to those of the bromo-bipyridine starting material. A principal difference is due to the inclusion of the methyl resonance at the aliphatic region of the spectrum. The ^13^C-NMR spectrum gave the anticipated peaks at 169.76, 156.21, 149.98, 137.86, 136.88, 129.74, 124.81, 121.32, 14.81 and 12.71. The bipyridine peaks due to chemical equivalency were observed in the range 156–128 ppm. The peak at 169.76 ppm was assigned to the carbonyl carbon; the two peaks at 124.81 and 121.32 were assigned to the alkenyl carbons, while the methyl groups were found at 14.81 and 12.71 ppm. The ^1^H-NMR of **L_2_** show five signals at the aromatic region at δ 9.20 (d), 8.57 (dd), 8.26 (d), 8.24 (d), 7.62 (dd) were assigned to the bipyridine and anthracene protons. The two singlet peaks at the aliphatic region were assigned to the *trans*- methyl protons of the carboxylic acid group at δ 2.17 and 1.67 ppm. The ^13^C spectrum of **L_2_** was similar to that of **L_1_**, except for those additional peaks at 131.03, 128.25, 127.44 and 126.49 ppm that were assigned to the anthracenyl carbons signals.

**Figure 2:**
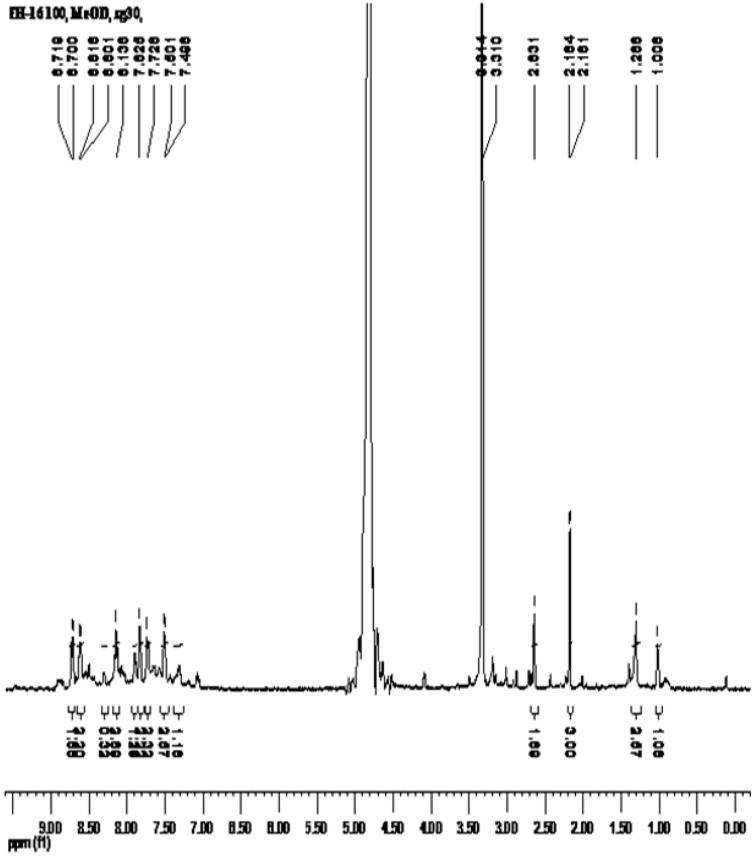
^1^H-NMR spectrum of [RuL_1_L_2_(NCS)_2_] in CD_3_OD.

The proton NMR spectrum of the [RuL_1_L_2_(NCS)_2_] complex in CD_3_OD (Figure 2) is consistent with the structure shown in Scheme 1. Due to the presence of two different anchoring ligand substitutions, such that all the protons are electronically found in different environments, gave complications in the spectrum most especially at the aromatic region. The spectrum showed six proton signals between δ 8.71-7.50 ppm integrating for twelve protons. The bipyridyl protons were observed as doublets at δ 8.71, 8.61 7.50 and a singlet at 8.14 ppm, while the anthracenyl protons were assigned to signals at δ 7.83 and 7.73 ppm. At the aliphatic region, the two conspicuous singlet peaks at δ 2.16 and 1.29 ppm, integrating for six protons due to chemical equivalence were unambiguously assigned to the methyl groups. In comparison to the individual ligands, the proton peaks in the complex were shifted downfield. The deshielding pattern is ascribed to the lone pair-lone pair electron donation of the nitrogen atoms to the *d*-orbital of the ruthenium metal. The ^13^C-NMR spectrum data of the complex could not be adequately assigned to individual carbon atoms due to poor signal resolutions.

### 2.4. Electronic Absorption and Emission Spectra

#### 2.4.1. Electronic absorption spectroscopy

**Figure 3:**
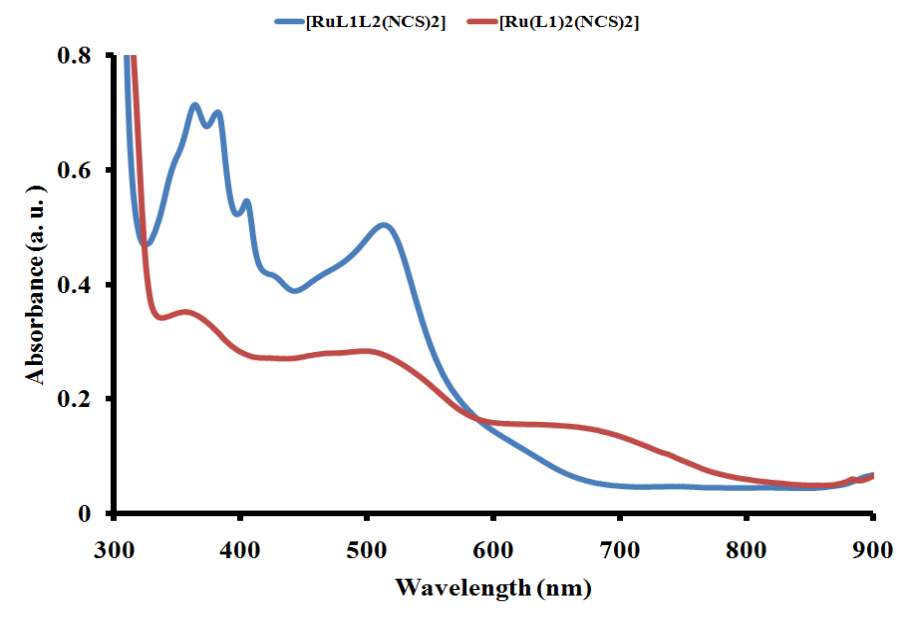
Comparison of the UV-Vis absorption spectrum of [RuL_1_L_2_(NCS)_2_] (blue line) and [Ru(L_1_)_2_(NCS)_2_] (red line) at a concentration of 1 × 10^−3^ M^−1^ in DMF solution showing effect of extended p-conjugation using anthracene.

**Figure 4:**
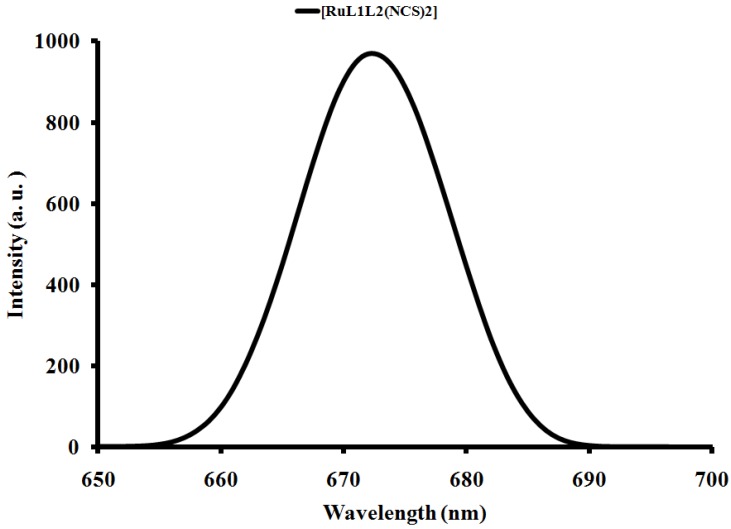
Emission spectrum of [RuL_1_L_2_(NCS)_2_] in DMF solution.

The UV-Vis absorbance and emission spectra of complex [RuL_1_L_2_(NCS)_2_] in dimethylformamide are shown in Figure 3 and Figure 4. In the UV-region, [RuL_1_L_2_(NCS)_2_] displays four distinct vibronic peaks for the intra ligand (π→π*) charge transfer transitions characteristics of anthracene derivatives at 366, 383, 406 and 430 nm. The complex shows broad and intense absorption bands between 419 and 600 nm with wavelength maxima at 518 nm. This absorption band was due to the metal-to-ligand charge transfer transition (MLCT) of the complex with the molar extinction coefficient (ε = 44900 M^−1^ cm^−1^). The effect of extending the π-conjugation length through anthracene was observed when [RuL_1_L_2_(NCS)_2_] complex spectrum was compared to a similar [Ru(L_1_)_2_(NCS)_2_] complex with no anthracene substitution (Figure 3). The molar extinction coefficient of the lowest energy MLCT band in the [Ru(L1)_2_(NCS)_2_] complex at 508 nm (ε ≈ 20350 M^−1^ cm^−1^) which is 45.3% lower than that of the [RuL_1_L_2_(NCS)_2_] complex. However, it terms of increase in the number of anthracenyl units, molar extinction was found to decrease proportionally as previously reported by us for two phenanthroline ruthenium(II) complexes. Thus it could be taken that the distance transfer of electronic energy through anthracene may have been hindered due to molecular aggregation [28,35]. Large pi-aromatic molecules, such as prophyrins, phthalocyanines, and perylenes have been reported to be important classes of potential sensitizers for highly efficient dye-sensitized solar cells, owing to their photostability and high light-harvesting capabilities that can allow applications in thinner, low-cost dye-sensitized solar cells [36].

#### 2.4.2. Emission study

The emission spectrum of complex [RuL_1_L_2_(NCS)_2_] is displayed in Figure 4. Upon excitation into the ^1^LC and ^1^MLCT bands (λ_exc_ = 500 nm), the complex displays appreciable luminescence at room temperature. An emission wavelength maximum was found at 673 nm. A direct correlation has been found between emission intensity and the molecular weight of compounds [35]. It is well known that conjugated functional organic molecules are useful for the study of electron transport at the molecular scale and that the use of fused-ring systems is a powerful and practical approach. It could be seen in the complex that the choice of ligand has significant effects on the energy ordering of the low energy excited state and, in particular to the orbital nature of the lowest excited state which also influence the positions of the metal centre (MC), ligand centre (LC) as well as the MLCT of the complexes. The energy of the MC excited state depends on the ligand field strength, which in turn depend on the σ-donor and π-acceptor properties of the ligands, the steric crowding around the metal (that can preclude a sufficient close approach between metal and ligand) [37,38,39], and the bite angle of the polydentate ligands (which in some cases cannot be optimized because of molecular constraints [40]. It has been shown that the energy of the MLCT excited state depends on the reduction potential of the ligand involved in the MLCT transition, the oxidation potential of the metal in the complex (which is affected by the electron donor and acceptor properties of all the ligands), and by the charge separation caused by the transition. The energy of the LC excited states depends on intrinsic properties of the ligands, such as the HOMO-LUMO energy gap and the singlet-triplet splitting. The intense emission is a significant contribution to the excited state from an interaction between the metal d-orbital and the ligand π-systems [41].

### 2.5. Electrochemical Study

**Figure 5:**
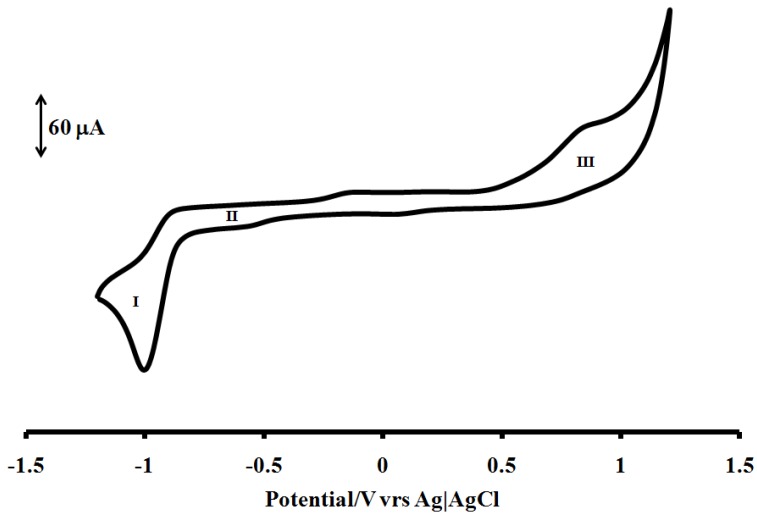
Cyclic voltammogram for [RuL_1_L_2_(NCS)_2_] complex at 1 × 10^−3^ M in freshly distilled DMF containing 0.1 M TBABF_4_ supporting electrolyte. Step potential = 5 mV, amplitude = 50 mV *vs.* Ag|AgCl, frequency = 10 Hz. Scan rate = 100 m Vs^−1^ *vs.* Ag|AgCl.

The cyclic voltammogram of [RuL_1_L_2_(NCS)_2_] complex in DMF, containing 0.1 M tetrabutyl-ammonium tetrafluoroborate as supporting electrolyte and Ag|AgCl electrode was studied in the potential range +1.5 to −1.5 V and at a scan rate 50 mV s^−1^ is shown in Figure 5. The voltammogram displays an irreversible oxidation peak (process **III)** at 0.84 V, this was assigned to the metal centre Ru(III)/Ru(II) couple. The oxidation potential of the complex [RuL_1_L_2_(NCS)_2_] is close to that of Ru^2+^ as reported by Grätzel and co-workers for other thiocyanate containing ruthenium(II) dyes [6]. The redox wave of the complex [RuL_1_L_2_(NCS)_2_] were observed at E_1/2_ = −0.95 and −0.53 V for processes **I** and **II**, respectively. The electrochemical properties of these types anthracenyl containing Ru(II) polypyridine complexes have been shown to possess good electroredox properties and that the redox processes could be both single- and multi- electronic in nature due to the incorporation of anthracene moiety [28]. By comparison, the number of electrons transfer by [RuL_1_L_2_(NCS)_2_] complex in the three redox processes were determined accurately by chronocoulometry method with the equation:

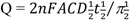



**Figure 6:**
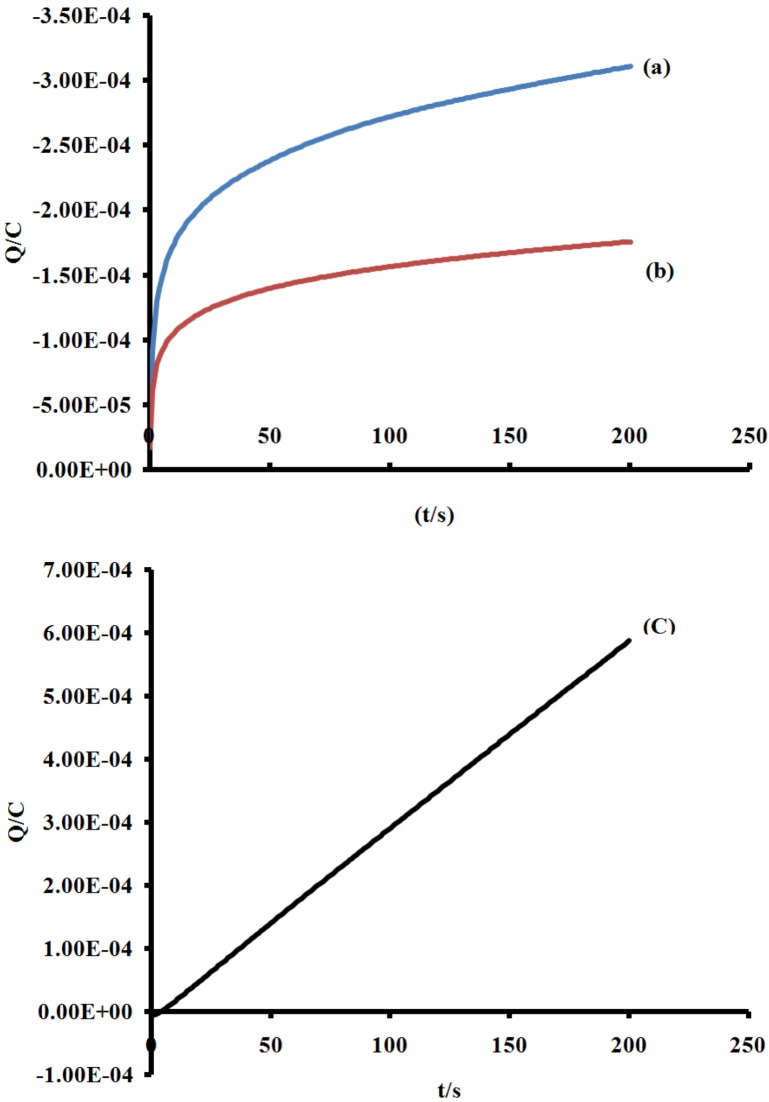
Plots of charge *vs*. time response for processes **I**, **II** and **III**; (line a–c, respectively). Scan rate = 200 m Vs^−1^.

**Figure 7:**
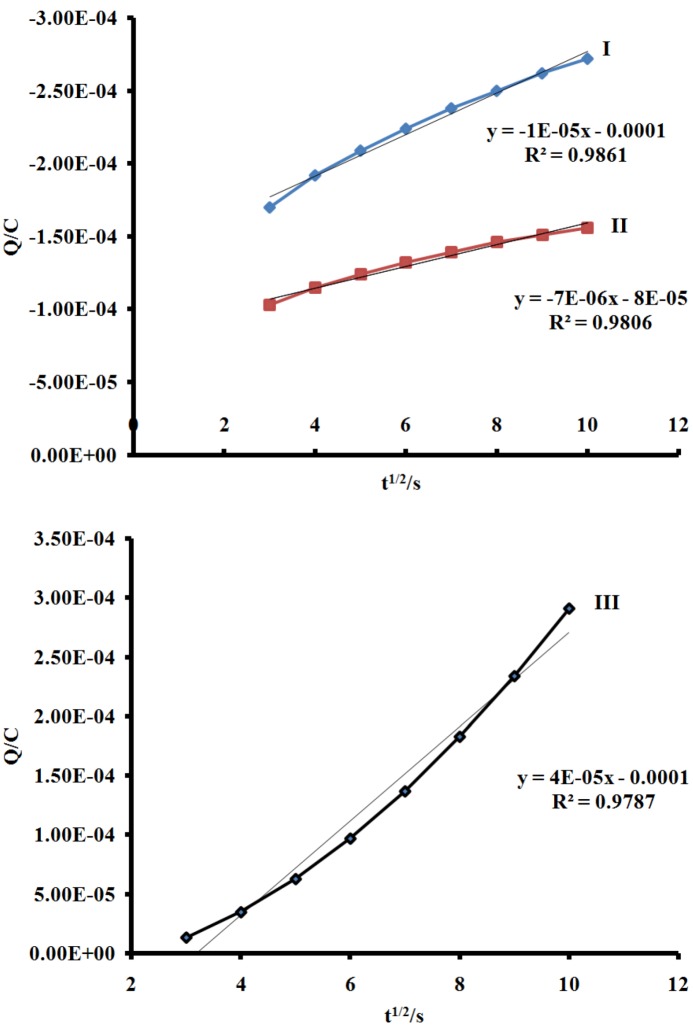
Anson plot of charge *vs*. square root of time (s) for processes I, II and III. Scan rate = 200 m Vs^−1^.

From the slope of the data when the quantity of electricity was plotted against square root of time (Figure 6 and Figure 7), the number of electrons (n) for processes **I**, **II** and **III** were found to be in ratio (1:1:1), suggesting that a single- electron process occurred in the redox wave characteristics of the molecule unlike the ruthenium(II) phenanthrolyl complex containing oligo-anthracenyl moieties [28].

## 3. Experimental

### 3.1. Materials and General Physical Measurements

All chemical and reagents were analytically pure and used without further purification. 4-Bromo-2,2'-bipyridine was synthesized as described in the literature [30]. 4-(2,3-Dimethylacrylic acid)-2,2'-bipyridine (**L_1_**) and 4-(9-anthracenyl-10-(2,3-dimethylacrylic acid)-2,2'-bipyridine (**L_2_**) were synthesized by the literature procedure [28] with slight modifications (Scheme 1). All thin layer chromatography (TLC) analyses were done with aluminium sheet precoated with normal phase silica gel 60 F_254_ (Merck, 0.20 mm thickness) unless otherwise stated. The TLC plates were developed using any of the following solvent systems: Solvent system A: Dichloromethane-Methanol (9:1); Solvent system B: Dichloromethane-Methanol (7:3); Solvent system C: Dichloromethane-Benzene (1:1); Solvent system D: Diethyl ether-Methanol (1:1). Gel filtration was performed using Sephadex LH-20 previously swollen in specified solvent (s) prior to loading of extract onto the column (3.5 cm × 8.5 cm).

Melting points were determined using a Gallenkamp electrothermal melting point apparatus. Microanalyses (C, H, N, and S) were carried out with a Fisons elemental analyzer and Infrared spectra were obtained with KBr discs or nujol on a Perkin Elmer System 2000 FT-IR Spectrophotometer. UV-Vis and fluorescence spectra were recorded in a 1 cm path length quartz cell on a Perkin Elmer Lambda 35 spectrophotometer and Perkin Elmer Lambda 45 spectrofluorimeter, respectively. ^1^H- and ^13^C-NMR spectra were run on a Bruker EMX 400 MHz spectrometer for ^1^H and 100 MHz for ^13^C. The chemical shift values were reported in parts per million (ppm) relative to (TMS) as internal standard. Chemical shifts were reported for the ligands with respect to CDCl_3_ at δc 77.00 and δ_H_ CDCl_3_ at 7.25; DMSO-d_6_ at δc 40.98 and DMSO-d_6_ at δ_H_ 2.50. The complex was run in CD_3_OD at δ_H_ 3.31. Electrochemical experiment was performed using a PGSTAT 302 Autolab potentiostat (EcoChemie, Utrecht, The Netherlands) driven by the general purpose Electrochemical System data processing software (GPES, software version 4.9). A conventional three-electrode system was used. The working electrode was a bare glassy carbon electrode (GCE), Ag|AgCl wire and platinum wire were used as the pseudo reference and auxiliary electrodes, respectively. The potential response of the Ag|AgCl pseudo-reference electrode was less than the Ag|AgCl (3 M KCl) by 0.015 ± 0.003 V. Prior to use, the electrode surface was polished with alumina on a Buehler felt pad and rinsed with excess millipore water. All electrochemical experiments were performed in freshly distilled dry DMF containing TBABF_4_ as supporting electrolyte.

### 3.2. Synthesis of 4-(2,3-DMAA)-2,2'-bipyridine *(**L_1_**)*

4-Bromo-2,2'-bpy (1.05 g, 3.38 mmol) and 2,3-dimethylacrylic acid (DMAA, 0.34 g, 3.38 mmol) were dissolved in MeOH (40 mL) in a 250 mL flask. Et_3_N (1.0 mL) and palladium-carbide (0.050 g) were added and the mixture was reflux for 8 h at a temperature between 110-120 °C. The reaction was allowed to cool to room temperature and the solvent removed under reduced pressure. The residue was dissolved in degassed water and then extracted with chloroform. The chloroform extract was concentrated *in vacuo* to obtain a brilliant colourless liquid which solidified after about 48 h at room temperature. The resultant residue was recrystallized from Et_2_O (30 mL). Colour: White crystalline solid; Percentage yield: 0.90 g, 67%, Melting point: ND; IR (KBr, υ_cm_^−1^): 3054, 2927, 2676, 1965, 1690, 1648, 1581, 1559, 1456, 1419, 1346, 1251, 1141, 1089, 1040, 992, 893, 757, 653, 631, 619, 555. ^1^H-NMR (DMSO): δ 8.66 (d, *J* = 4.0 Hz, H-6, 6’), 8.41 (d, *J* = 8.0 Hz, H-3, 3’), 7.86 (dd, *J* = 7.6, 8.0 Hz, H-5, 5’), 7.36 (dd, *J* = 5.2, 7.2 Hz, H-4’), 1.73 (s, CH_3_), 1.66 (d, CH_3_). ^13^C-NMR (DMSO): δ 169.76, 156.21, 149.98, 137.86, 136.88, 129.74, 124.81, 121.32, 14.81, 12.71. Elemental Analysis: Calculate H 5.55, C 70.85, N 11.02; required H 5.55, C 70.60, N 11.43. Molecular Formula: C_15_H_14_N_2_O_2_.

### 3.3. Synthesis of 4-(9-Anthracenyl-10-(2,3-DMAA))-2,2'-bipyridine *(**L_2_**)*

4-Bromo-2,2'-bpy (1 g, 4.82 mmol) and 9-bromo-10-(2,3-dimethylacrylic acid)-anthracene (1.72 g, 4.82 mmol) were dissolved in benzene-dichloromethane (50 mL, v/v, 1:1), followed by the addition of Et_3_N (1 mL), KOH (0.27 g, 4.82 mmol) and palladium-carbide (0.05 g). The reaction was carried out under reflux for 12 h at temperature 110-120 °C. Colour: Yellow crystalline solid. Melting point: 167-169 °C, Percentage yield: 1.93 g, 71%, IR (KBr): 3427, 3056, 2926, 1952, 1802, 1690, 1622, 1582, 1558, 1524, 1456, 1437, 1420, 1349, 1304, 1256, 1162, 1149, 1089, 1040, 1028, 995, 926, 747, 676, 654, 619, 605, 578. ^1^H-NMR (DMSO): δ 9.20 (2d, *J* = 1.6, 4.4 Hz, H-6, 6'), 8.57 (dd, *J* = 3.2, 6.8 Hz, H-a, b), 8.26 (d, *J* = 2.0 Hz, H-3, 5), 8.24 (d, *J* = 1.6 Hz, H-4'), 7.62 (dd, *J* = 3.2. 6.8 Hz, H-c, d), 2.17 (s, CH_3_), 1.67 (s, CH_3_). ^13^C-NMR (DMSO): δ 156.76, 150.31, 146.27, 144.21, 135.94, 131.03, 128.52, 128.25, 127.44, 126.49, 124.21, 123.51, 123.04, 30.90 and 21.92. Elemental Analysis: Calculate H 5.15, C 80.91, N 6.51; required H 5.51, C 80.67, N 6.23. Molecular Formula: C_29_H_22_N_2_O_2_.

### 3.4. Synthesis of cis-Dithiocyanato-4-(2,3-dimethylacrylic acid)-2,2'-bipyridyl-4-(9-anthracenyl-(2,3-dimethylacrylic acid)-2,2'-bipyridyl-ruthenium(II) complex ***[RuL_1_L_2_(NCS)_2_]***

In a 250 mL flask, [RuCl_2_(dmso)_4_] (0.24 g, 0.49 mmol) was dissolved in *N,N*-dimethyl-formamide/MeOH (80 mL, 1:1, v/v) followed by the addition of ligand **L_1_** (0.25 g, 0.98 mmol) and **L_2_** (0.42 g, 0.98 mmol). The mixture was refluxed initially at 120 °C for 2 h in the dark and excess of NH_4_NCS (1.50 g, 1.95 mol) was added. The reaction was left to reflux for 10 h. The solution was allowed to cool to room temperature and then filtered to remove unreacted starting material. The filtrate was concentrated to dryness and 40 mL of 0.05 M NaOH solution was added to give a dirty brown precipitate which was filtered off. The pH of the resulting solution was adjusted to 3 with 0.5 M HNO_3_. The solution was left to stand in the fridge (−2 °C) for 12 h before being filtered and concentrated *in vacuo*. The crude residue product was adsorbed onto Sephadex LH-20 adsorbent in a glass column and eluted using solvent system D (diethyl ether-methanol, 50%, v/v, 250 mL). Colour: Dark brown solid, Melting point: >260 °C, Percentage yield: 0.60 g, 36%. IR (KBr) ν_max_/cm^−1^: 3551, 3478, 3414, 3239, 3066, 2922, 2103, 1635, 1617, 1584, 1533, 1459, 1443, 1419, 1309, 1239, 1155, 1089, 1039, 1014, 961, 926, 839, 805, 760, 718, 682, 619, 611, 557, 473, 426, 399. UV-Vis (λ_max_/nm, ε = M^−1^ cm^−1^, DMF): 366 (64000), 383 (62800), 406 (48650), 430 (36977), 518 (44900). Emission wavelength: (λ_exc._ = 500 nm, λ_em_ = 680 nm). ^1^H-NMR (DMSO-d_6_): δ 8.71 (d, *J* = 7.6 Hz, 2H), 8.61 (d, *J* = 6.8 Hz, 2H), 8.14 (s, br, 2H), 7.83 (br, 2H), 7.73 (br, 2H), 7.50 (d, *J* = 1.2 Hz, 2H), 2.16 (s, 3H), 1.29 (s, 3H). ^13^C-NMR (CDCl_3_): ND. Cyclic voltammetry Data: Ru^2+^/Ru^3+^ = 0.84 V; E_cathodic_ = −0.53 V, E_1/2_ = −0.95 V. Elemental Analysis: Calculate H 4.02, C 61.25, N 9.32, S 7.11; required H 4.43, C 61.51, N 9.66, S 7.57. Molecular Formula: RuC_46_H_36_N_6_O_4_S_2. _

## 4. Conclusions

This work has reported the synthesis and characterization of two new 4-monosubstituted 2,2'-bipyridine ligands and the corresponding heteroleptic ruthenium(II) complex. The photophysical, spectroscopic and electroredox properties of the complex were studied. One of the ligands in particular, with a substituted anthracene derivative, was introduced to function as a source of increased π-conjugative bonds in the complex, with a view to enhancing the molar extinction coefficient of the molecule. The complex showed enhanced and better photophysical, photoluminescence and electrochemical properties that are common to ruthenium(II) polypyridine complexes. However, in comparison to our previous observations towards the study of a systematic increase in the number of anthracene units in the ruthenium(II) phenanthroline complexes, the UV-Vis absorption and luminescence property of this complex revealed that extension of the π-bond conjugation on these types of complexes may not be the only contributory factor responsible for the shift in absorption wavelengths to the red region, with corresponding increase in the molar absorptivity coefficient, but the types, position of substituents on ligands, use of heteronuclear ligands and/or the molecular weight of compounds could as well be very vital or the determining factor in the overall characteristics displayed by the complexes.

Particularly for this molecule, further work is ongoing in our laboratory to establish the solar-to-electrical energy conversion efficiency (η) in the dye-sensitized solar cells (DSSCs). However, due to π-electron cloud overlaps in anthracene derivatives, the molecule could find use in applications, including organic electroluminescence material for biosensitizers, and display devices such as Organic Light Emitting Diode (OLED), Organic Thin Film Transistor (OTFT), wearable display, photochromic agents and electronic paper.

## Supplementary Materials

Supplementary materials can be accessed at http://www.mdpi.com/1420-3049/16/6/4615/s1.
